# Early identification of sepsis in hospital inpatients by ward nurses increases 30-day survival

**DOI:** 10.1186/s13054-016-1423-1

**Published:** 2016-08-05

**Authors:** Malvin Torsvik, Lise Tuset Gustad, Arne Mehl, Inger Lise Bangstad, Liv Jorun Vinje, Jan Kristian Damås, Erik Solligård

**Affiliations:** 1 Faculty of Health Science, Nord University, Høgskoleveien 27, N-7600 Levanger, Norway; 2 Department of Internal Medicine, Levanger Hospital, Nord-Trøndelag Hospital Trust, Kirkegata 2 A, N-7600 Levanger, Norway; 3 Department of Neuroscience, NTNU, Norwegian University of Science and Technology, Edvard Griegs gate 9, N-7030 Trondheim, Norway; 4 Mid-Norway Sepsis Research Group, Faculty of Medicine, NTNU, Norwegian University of Science and Technology, Trondheim, Norway; 5 Unit for Applied Clinical Research, Department of Cancer Research and Molecular Medicine, NTNU, Norwegian University of Science and Technology, Prinsesse Kristinas gate 1, N-7030 Trondheim, Norway; 6 Centre of Molecular Inflammation Research, Department of Cancer Research and Molecular Medicine, NTNU, Norwegian University of Science and Technology, Prinsesse Kristinas gate 1, N-7030 Trondheim, Norway; 7 Department of Infectious Diseases, St Olavs Hospital, Trondheim University Hospital, Olav Kyrres gate 17, N-7030 Trondheim, Norway; 8 Clinic of Anesthesia and Intensive Care, St Olavs Hospital, Trondheim University Hospital, Olav Kyrres gate 17, N-7030 Trondheim, Norway; 9 Department of Circulation and Medical Imaging, NTNU, Norwegian University of Science and Technology, Olav Kyrres gate 17, N-7030 Trondheim, Norway

**Keywords:** Systemic inflammatory response syndrome, Sepsis, In-hospital, Adherence, Survival

## Abstract

**Background:**

Systemic inflammatory response syndrome (SIRS) and sepsis are now frequently identified by observations of vital signs and detection of organ failure during triage in the emergency room. However, there is less focus on the effect on patient outcome with better observation and treatment at the ward level.

**Methods:**

This was a before-and-after intervention study in one emergency and community hospital within the Mid-Norway Sepsis Study catchment area. All patients with confirmed bloodstream infection have been prospectively registered continuously since 1994. Severity of sepsis, observation frequency of vital signs, treatment data, length of stay (LOS) in high dependency and intensive care units, and mortality were retrospectively registered from the patients’ medical journals.

**Results:**

The post-intervention group (n = 409) were observed better and had higher odds of surviving 30 days (OR 2.7, 95 % CI 1.6, 4.6), lower probability of developing severe organ failure (0.7, 95 % CI 0.4, 0.9), and on average, 3.7 days (95 % CI 1.5, 5.9 days) shorter LOS than the pre-intervention group (n = 472).

**Conclusions:**

In a cohort with stable mortality rates, early sepsis recognition by ward nurses may have reduced progression of disease and improved survival for patients in hospital with sepsis.

**Electronic supplementary material:**

The online version of this article (doi:10.1186/s13054-016-1423-1) contains supplementary material, which is available to authorized users.

## Background

The Surviving Sepsis Campaign was launched in 2004 as a global initiative to improve survival and reduce the morbidity associated with sepsis [1–3]. Rapid response systems and development of care bundles have been central to the initiative [4]. However, triggering of evidence-based treatment systems requires recognition of sepsis and the initiation of a treatment response as early as possible [5]. For the last two decades, sepsis has been defined as suspected or evident infection accompanied by at least two signs of systemic inflammatory response syndrome (SIRS) [6]. Observations of patients with suspected infection, including vital signs and organ function, have been insufficiently monitored for development of sepsis, especially at ward level [5]. Ward nurses, who are at the bedside of patients in hospital, are in a key position to identify early-stage sepsis and development of organ failure, yet they have not been central to the sepsis campaign [7]. It is not known how a systematic continuation of observations of SIRS and organ failure in hospitalized patients with suspected or confirmed infection will impact the outcomes of patients with sepsis. Within the Mid-Norway Sepsis Study, the rich data source on observations, treatment and outcome in patients with confirmed bloodstream infection (BSI) makes it possible to assess the potential effects of such improvements in the treatment chain.

The aim was to investigate whether implementation of a clinical tool for triage of SIRS and organ failure at the ward, an alert and treatment flow chart, reinforced by training, could improve clinical observations, lead to fewer patients developing severe sepsis, and thus improve in-hospital survival among patients with BSI.

## Methods

### Study population

Since 1994 the Mid-Norway Sepsis Study has continuously prospectively registered patients with confirmed BSI identified by growth of one or more microbes from blood culture, combined with clinical evidence of systemic infection [8]. A new occurrence of BSI was recorded for patients if at least 30 days had passed since a previous incident [9]. Clinical information from the patients’ records was recorded retrospectively, after standardized definitions, by a team of trained health care professionals for all patients admitted before 2014 [8].

### Intervention

**Fig. 1:**
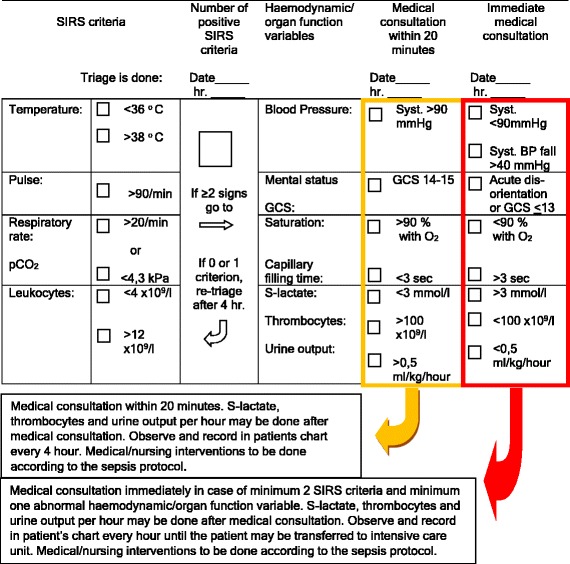
Systemic inflammatory response syndrome (*SIRS*) and organ failure triage (SOF-Triage), which should be used for all inpatients with suspected infection, and clinical indication for monitoring. *GCS* Glasgow coma scale

The intervention was implemented from January to October 2011 in one emergency community hospital, serving around 90,000 citizens, in the Mid-Norway Sepsis Study catchment area. The hospital has 124 beds, approximately 15,700 admissions annually, and has 6 beds in the intensive care unit (ICU) and 5 in the high dependency unit (HDU), equivalent to 12.6 ICU and HDU beds/100,000 citizens. The mean patient load across all staff-registered nurses who are involved in bedside work is 0.24 [10]. The hospital does not have a formal Critical Care Outreach service, but there is daily dialog between the ward physician and the ICU doctors to discuss patients in possible need of transfer to the ICU/HDU. Acute transfers are handled by the ICU doctor on call. The intervention was a bundle that included a flow chart for sepsis identification, treatment and physician response time (see Additional file 1), a SIRS and organ failure triage (SOF-Triage) (Fig. 1) that was used on the wards to re-evaluate for sepsis if clinically indicated. The new flow chart instructed the ward nurse in charge of the sepsis patient to call the ICU doctor on call directly if the ward physician was occupied and prevented from applying within the time limits of the SOF-Triage. Additionally, the bundle included information to all physicians and a new four-hour training course for all nurses and nursing students working on the wards (medical, surgical and gynecological). The training included the pathophysiology, early signs, and treatment of sepsis. The latter included the importance of prompt intravenous (i.v.) fluids and appropriate antibiotics, training in the SOF-Triage, and objective communication about the patients’ status based on observation of vital signs. Six extra training sessions (on the background of the project, interpretation of blood gas analysis, use of the SOF-Triage and flow chart) was given to twelve expert nurses (at least one nurse in each ward), who assisted in the implementation of the intervention.

The pre-intervention group included patients with confirmed BSI in the period from January 2008 to December 2010 as this period had guidelines that mandated equal vigilance towards observation of vital signs as the intervention group (respiratory frequency, heart rate, and temperature should be observed at least every 4 hours per first 24 hours in patients with suspected sepsis). The post-intervention group consisted of patients with BSI admitted after implementation of the intervention, i.e., from Nov. 2011 to Dec. 2013.

### Data collection

The variables were categorized as follows: the observation-rate of each vital sign the first 24 hours after drawing a blood culture (temperature, respiratory frequency or heart rate) was graded as poor (0 or 1 recorded observation), some (2–4 recorded observations) or good observation (≥5 recorded observations); sepsis at admission as BSI without signs of SIRS, sepsis, severe sepsis, or septic shock [6]; sepsis-related Sequential Organ Failure Assessment (SOFA score) [11] at baseline and during follow up was dichotomized as non-severe or severe organ failure (corresponding to infection-related SOFA score >2 in any organ) [8]; infection focus as lungs, urinary tract, and other origin; BSI-type as gram-negative, gram-positive, and mixed microbial BSI (Additional file 2); length of stay (LOS) in the HDU and ICU and survival were counted in days, 0–24 hours was coded as 1 day, >24–48 hours as 2 days and so forth. All patients were censored after 30 days of observation; age (in years) at the time of positive blood culture was treated as a continuous variable and as a categorical variable (<65 years, 65–79 years, and >80 years); McCabe score was used to exclude patients with rapidly fatal illness at admission (death expected within 1 month) [12]; the Charlson weighted Comorbidity Index (CCI) was classified as low (no underlying disease score), medium (score 1–2), or high (score >2) [13]; place of acquisition was categorized as hospital-acquired if a positive blood culture was drawn >48 hours after admission, healthcare-associated (HCA) or community-acquired as defined by Friedman et al. [14], with the exception that HCA infection was defined for patients hospitalized for ≥2 days in the last 30 days (instead of the last 90) before admission, as recommended by Schorr et al. [15]; functional status was categorized as independent if the patient lived at home without any help from the community health service, partly independent if the patient had some help from the community health service, e.g., with medication or wound care, and dependent if the patient lived in a nursing home or lived at home with help in most of the daily life activities [8]; appropriate empiric antibiotic therapy was defined if (1) given intravenously in correct doses within 24 hours after the blood culture and (2) active in vitro against the isolated microbe(s) [16]; intravenous fluid was documented as amount in mL given during the first 24 hours after diagnosis.

## Statistical analysis

We excluded all patients with rapidly fatal illness from the analysis. The *t* test for continuous variables, and χ^2^ test for categorical data were used to compare patient baseline characteristics and nurses’ observation in pre-intervention and post-intervention groups. The *t* test was used to find the mean difference ± SD (95 % CI) between groups, and general linear models (GLM) to find differences between groups adjusted for covariates in four different models; unadjusted (model 1), age (continuous variable) and sex (model 2), model 2 plus functional status and Charlson Comorbidity Index (model 3) and model 3 plus dichotomized SOFA score (model 4). The χ^2^ test was used to examine the proportions of patients who developed severe organ failure during the hospital stay amongst patients admitted without a severe SOFA score in pre-intervention and post-intervention groups, and to examine the difference in the numbers of patients who were deceased by 7 and 30 days. Logistic regression analyses with the four previously described models were used to calculate the odds ratios (ORs) and 95 % CIs for surviving 7 and 30 days after positive blood culture (the pre-intervention group served as the reference group). In additional survival analysis, we examined these variables added one by one to model 4: age categories, place of acquisition, use of immunosuppression, and focus of infection.

In sensitivity analyses, all the above analyses were repeated restricted to first episodes of sepsis. Finally, we investigated the potential effect of modifications by sex and age (dichotomized at age 65 and age 80 years). Analyses were performed with STATA SE/13.1 12 © StataCorp LP.

## Results

### Study population

A total of 478 BSI patients were admitted in the pre-intervention period and 422 in the post-intervention period. We excluded 19 patients with rapidly fatal illness, 6 (1.26 %) in the pre-intervention group and 13 (3.1 %) in the post-intervention group (*p* = 0.001). Thus, the pre-intervention group included 472 BSI episodes, and the post-intervention group included 409 BSI episodes. The post-intervention group had a higher proportion of patients with a severe SOFA score and the patients were less in need of help in daily life than in the pre-intervention group. For more details about the groups see Table 1. The ratios of gram-negative, gram-positive and mixed microbial BSI were similar in the two groups as Table 1 shows.

**Table 1:** Baseline characteristics at the time of drawing the first positive blood culture in the pre-intervention and post-intervention group (n = 881)

	Pre-intervention	Post intervention	
	(n = 472)	(n = 409)	
Variables	Number (%)	Number (%)	*P*
Age			0.340
< 65 years	147 (31.1)	127 (31.1)	
65– ≤ 80 years	150 (31.8)	147 (35.9)	
> 80 years	175 (37.1)	135 (33.0)	
Female	232 (49.2)	218 (53.3)	0.219
Place of acquisition			0.201
Community-acquired	216 (45.8)	164 (40.1)	
Healthcare-acquired	195 (41.3)	192 (46.9)	
Hospital-acquired	61 (12.9)	53 (13.0)	
Functional status			<0.001
Independent	269 (57.2)	284 (69.4)	
Partly independent	139 (29.6)	72 (17.6)	
Dependent	62 (13.2)	53 (13.0)	
Charlson Comorbidity Index			0.624
0	123 (26.1)	95 (23.2)	
1–2	186 (39.4)	167 (40.8)	
≥ 3	163 (34.5)	147 (36.0)	
Infection severity			<0.001
BSI without sepsis	21 (4.4)	2 (0.5)	
Sepsis	352 (74.6)	274 (67.0)	
Severe sepsis	90 (19.1)	123 (30.1)	
Septic shock	9 (1.9)	10 (2.4)	
SOFA score >2 in any organ	97 (20.6)	132 (32.3)	
Infection focus			0.370
Lungs	73 (15.5)	59 (14.4)	
Urinary tract	189 (40.0)	183 (44.8)	
Other and unknown	210 (44.5)	167 (40.8)	
Bloodstream infection (BSI) categories			0.741
Gram-negative BSI	263 (55.7)	232 (56.7)	
Gram-positive BSI	178 (37.7)	146 (35.7)	
Mixed microbial	31 (6.6)	31 (7.6)	
Antibiotic before admission (yes)	64 (13.6)	71 (17.4)	0.118
Appropriate antibiotic therapy within 24 h	402 (85.2)	356 (87.0)	0.424
Immunosuppressant use (yes)	69 (14.6)	73 (17.9)	0.194

*SOFA* severe organ failure (score >2 in any organ using the Sequential Organ Failure Assessment) at the time of diagnosis, *BSI* bloodstream infection

### Observation and treatment of the patients

**Fig. 2:**
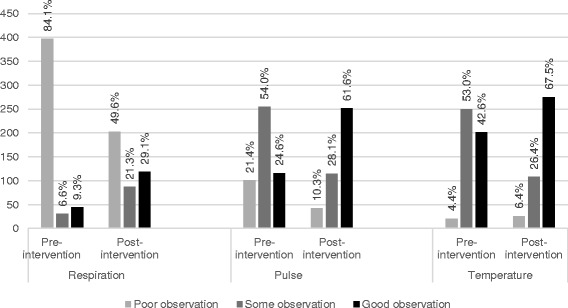
Nurses’ adherence to guidelines for each observation of vital signs (temperature, heart rate or respiratory frequency) during the 24 hours after drawing the first positive blood culture from patients with bloodstream infection (n = 881). Poor observation = 0–1 observation, some observations = 2–4 observations, and good observation = ≥5 observations. The post-intervention group had better observations of all vital signs in patients both with and without organ failure (χ^2^ test: *p* ≤ 0.002 for all vital signs)

The nurses in the post-intervention group increased their observation frequency of all vital signs (see details in Fig. 2). The post-intervention group had better observations of all vital signs in both patients with and without organ failure (*p* ≤0.002 for all vital signs).

The pre-intervention and post-intervention group had the same probability of receiving appropriate antibiotics within 24 hours (*p* = 0.89). The post-intervention group received on average 429.6 mL (95 % CI 137.3, 722.0 mL) more i.v. fluid during the first 24 hours after sepsis diagnosis in the GLM model 1. This difference was attenuated after adjustment for dichotomized SOFA score in model 3 (159.7 mL, 95 % CI-114.3, 433.8 MI). The mean LOS in the HDU/ICU was 3.7 days (95 % CI 5.9, 1.5 days) shorter in the post-intervention group than in the pre-intervention group in the full GLM adjustment models (model 4).

### Patient outcomes

In total 375 (79.4 %) of the pre-intervention group had a non-severe SOFA score on admission compared to 277 (67.7 %) in the post-intervention group. During the hospital stay, more patients in the pre-intervention group had deterioration classified by SOFA score as severe (21.3 %) compared to the post-intervention group (15.4 %) (*p* value = 0.025 for comparison of the two groups). The OR (95 % CI) for deterioration of the SOFA score in the post-intervention group was 0.6 (0.4, 0.9) when the pre-intervention group served as the reference.

At 7 days after confirmed BSI, 22 patients (4.6 %) in the pre-intervention group had died compared to 14 (3.4 %) patients in the post-intervention group (*p* = 0.36). After 30 days 59 (12.5 %) patients in the pre-intervention group had died versus 29 (7.1 %) patients in the post-intervention group (*p* = 0.035). Table 2 displays the ORs (95 % CIs) for surviving 7 and 30 days after BSI diagnosis in the pre-intervention and post-intervention groups in the different adjustment models.

**Table 2:** Hospital inpatients’ odds of surviving 7 and 30 days after the time of drawing the first positive blood culture in the post-intervention group compared to the pre-intervention group (reference group) (n = 881)

	Model 1	Model 2	Model 3	Model 4
	OR (95 % CI)	OR (95 % CI)	OR (95 % CI)	OR (95 % CI)
Survival at 7 days	1.4 (0.7, 2.7)	1.4 (0.7, 2.7)	1.3 (0.7, 2.7)	1.7 (0.8, 3.4)
Survival at 30 days	1.9 (1.2, 3.0)	1.9 (1.2, 3.0)	1.8 (1.1, 2.9)	2.7 (1.6, 4.6)

Model 1: unadjusted model. Model 2: adjusted for age and sex. Model 3: Model 2 + functional status and Charlson Comorbidity Index. Model 4: Model 3 + dichotomized Sequential Organ Failure Assessment score

In additional adjustment analyses for the variables immunosuppression, focus of infection, place of acquisition, and age in categories attenuated the impact of the intervention from 2.7 towards 2.0 (95 % CI 1.2, 3.3), see Additional file 3 for the details.

On sensitivity analyses for the first incidents of sepsis (n = 738) there was a stronger effect on survival in the post-intervention group than if all episodes of BSI (n = 881) were included. The post-intervention group with the first incidents of BSI had an OR of 2.4 (95 % CI 1.0, 5.3) for surviving 7 days and an OR of 3.6 (95 % CI 1.9, 6.9) for surviving 30 days compared to the pre-intervention group. The additional analyses for the first incidents of sepsis are provided in Additional file 4 (baseline characteristics of first-time incidents of BSIs) and Additional file 5 (odds of surviving 7 and 30 days in the post-intervention group). We found no evidence of interaction between survival and age or sex in any of the analysis (*p* > 0.1 for all).

## Discussion

In this pre-intervention and post-intervention study of the implementation of a sepsis-specific triage, flow chart alert and treatment system for inpatients, there was increased 30-day survival, fewer patients deteriorating to severe sepsis, and shorter LOS in the HDU/ICU in the post-intervention group.

The increased survival in the post-intervention group is important as this has constantly been the primary goal of the surviving sepsis campaign [6, 17, 18]. These results must be interpreted with caution as in other cohorts, as sepsis-related mortality has been reported to decrease over time [19, 20] and may not be a result of the intervention. However, there was stable mortality risk *Staphylococcus aureus* and *Streptococcus pneumoniae* in the period 1996–2011 for both 30-day and 90-day mortality in the same population as our cohort [21, 22]. Decreased mortality in the post-intervention group is thus, in our opinion, best understood as being related to the intervention.

We may hypothesize that this effect was due to a combination of several factors, with increased knowledge amongst all staff, more rigorous protocols including training in time-critical reporting of information across health care professions, better adherence to sepsis bundles, higher awareness and enhanced performance amongst all team members in our study, evidenced by improved observation of patients by nurses [23–27]. Early recognition and prompt management has been hypothesized to prevent patients with suspected infection from progressing to life-threatening sepsis. However, earlier research has focused on patients with severe sepsis and septic shock [28–30]. Our study is, to our best knowledge, the first study that includes the effects of more rigorous re-evaluation of hospital inpatients with sepsis and confirmed BSI, and it confirms that fewer patients develop severe sepsis. Our SOF-Triage and flow chart may thus be valuable tools to evaluate those inpatients with suspected infection who do not meet the new sepsis criteria [31].

We also found beneficial outcomes in the LOS in the HDU/ICU. We do not believe that this is due to organizational factors in the hospitals, as an emphasis to keep the LOS as short as possible has been in place at least since 2006 in Norway [32]. LOS in the HDU/ICU indicates less need for advanced treatment, and may be the result of more adequate treatment on the ward, less delay in admission to the ICU/HDU, or both. There are higher costs associated with caring for patients on ICU and HDU than caring for ward patients [33], and thus, an easy and affordable intervention as described in this study will be cost-efficient in the long term. The study shows how important it is from the perspective of the safety of patients with BSI, to allocate resources for bacteremia registries to evaluate (1) how the quality of care impacts the morbidity and mortality of patients with sepsis, and (2) evaluate the impact of patient identification and treatment flow charts.

The SIRS criteria that were launched in 1991 have been under debate because of poor sensitivity and specificity for sepsis [34, 35], and are not feasible for stand-alone diagnosis of sepsis. Hence, our SOF-Triage was developed based on both SIRS and organ failure assessment, which also is in line with the approach to sepsis in the sepsis guidelines launched by The Third International Consensus Definitions for Sepsis and Septic Shock (Sepsis-3) [31]. In line with suggestions from earlier literature, we designed it to assist in the monitoring of sepsis development and also to help nurses communicate in a precise language when referring patients to the physicians before severe organ failure develops [36, 37]. Adherence to observation and treatment protocols seems to improve when followed up with organized training and supervision [38], which was confirmed in our study. The nurses in the post-intervention period were better at monitoring all vital signs, including observation of respiratory rate. In spite of the importance of continuous observation of respiratory rate to detect deterioration, this observation is often missing in nurse monitoring of patients [39].

The new quick SOFA (q-SOFA) criteria is a simple and a promising tool for identifying patients at risk of sepsis with life-threatening organ dysfunction [31]. It is important to note that many patients in our study, who were prevented from developing severe sepsis, would not have been recognized by the q-SOFA criteria. We believe the SOF-Triage may be a complementary tool for observing inpatients who are admitted with, or develop an infection during the hospitalization. The new sepsis consensus also stresses that the SIRS criteria remain useful for the identification of infection, and the practitioner’s clinical assessments should not lead to a deferral of investigation or treatment of infection, on failure to meet the required q-SOFA criteria or SOFA score [31].

## Strength and limitations

This study has its apparent strengths including inter-departmental and cross-professional agreement and commitment. Further, all patients diagnosed with evident bacteremia were included. Nevertheless, the study has important limitations such as the use of a historical pre-intervention group, which does not ensure comparability between pre-intervention and post-intervention groups. In our analyses, we have also controlled for all known differences between the groups at baseline. However, as this was an observational study we could not control for a natural decline in mortality over time.

The study included patients with evident bacteremia only. Thus, a considerable proportion of patients with sepsis may have been left out, as positive blood culture is found in only 30–40 % of patients with sepsis [31]. However, there is no reason to believe that the proportion of confirmed BSIs should be different in the pre-intervention and post-intervention group.

## Conclusion

A sepsis specific triage, flow chart alert and treatment system for inpatients where the ward nurses are responsible for being in the forefront of sepsis diagnosis, may lead to increased survival, decreased occurrence of severe sepsis/septic shock, and shorter LOS in the ICU/HDU. Implementation of clinical tools needs to be discussed within the professional team and be supported by training to improve clinical observations. Our study also contributes to the understanding of how infection among hospital inpatients, who do not have a score ≥2 in q-SOFA, but still have indications for clinical monitoring, should be followed up on the wards. Thus, the importance of ongoing sepsis registries and evaluation of innovations of flow charts for observation and treatment of patients with BSI is important.

## Key messages

- Implementation and training in the use of a SIRS and organ failure triage (SOF-Triage) together with a patient flow chart improved the observations of all vital signs in patients with and without organ failure, and is believed to help ward nurses in early identification of patients with sepsis
- Early recognition and prompt management may prevent patients with BSI from progressing to life-threatening sepsis, reduce mean LOS in the HDU/ICU and increase 30-day survival
- The SOF-Triage may identify hospital inpatients, who are in need of close monitoring, even though they do not yet have a score ≥2 in the new quick SOFA.

## Abbreviations

BSI, bloodstream infection; CCI, Charlson Comorbidity Index; HCA, healthcare associated; HDU, high dependency unit; ICU, intensive care unit; i.v., intravenous; LOS, length of stay; q-SOFA, quick SOFA; SIRS, systemic inflammatory response syndrome; SOFA, Sequential Organ Failure Assessment; SOF-Triage, systemic inflammatory response syndrome and organ failure triage

## Additional files

Additional file 1: Flow chart for sepsis identification, treatment and physician response time. (TIF 11739 kb) Additional file 2: Detailed bloodstream infection (BSI) categorization in the pre-intervention and post-intervention group. (DOCX 13 kb) Additional file 3: Sensitivity analysis for further potential confounders in the association between intervention and survival in all patients with BSI. (DOCX 12 kb) Additional file 4: Baseline characteristics of patients admitted with the first incident of bloodstream infection. (DOCX 13 kb) Additional file 5: Odds of surviving 7 and 30 days in the first episode of sepsis in the pre-intervention group compared to the post-intervention group. (DOCX 12 kb)
