# Supplementary figures and images for: Early identification of sepsis in hospital inpatients by ward nurses increases 30-day survival
Source: Crit Care. 2016 Aug 5;20:244. doi: 10.1186/s13054-016-1423-1 (PMC4974789; doi:10.1186/s13054-016-1423-1)

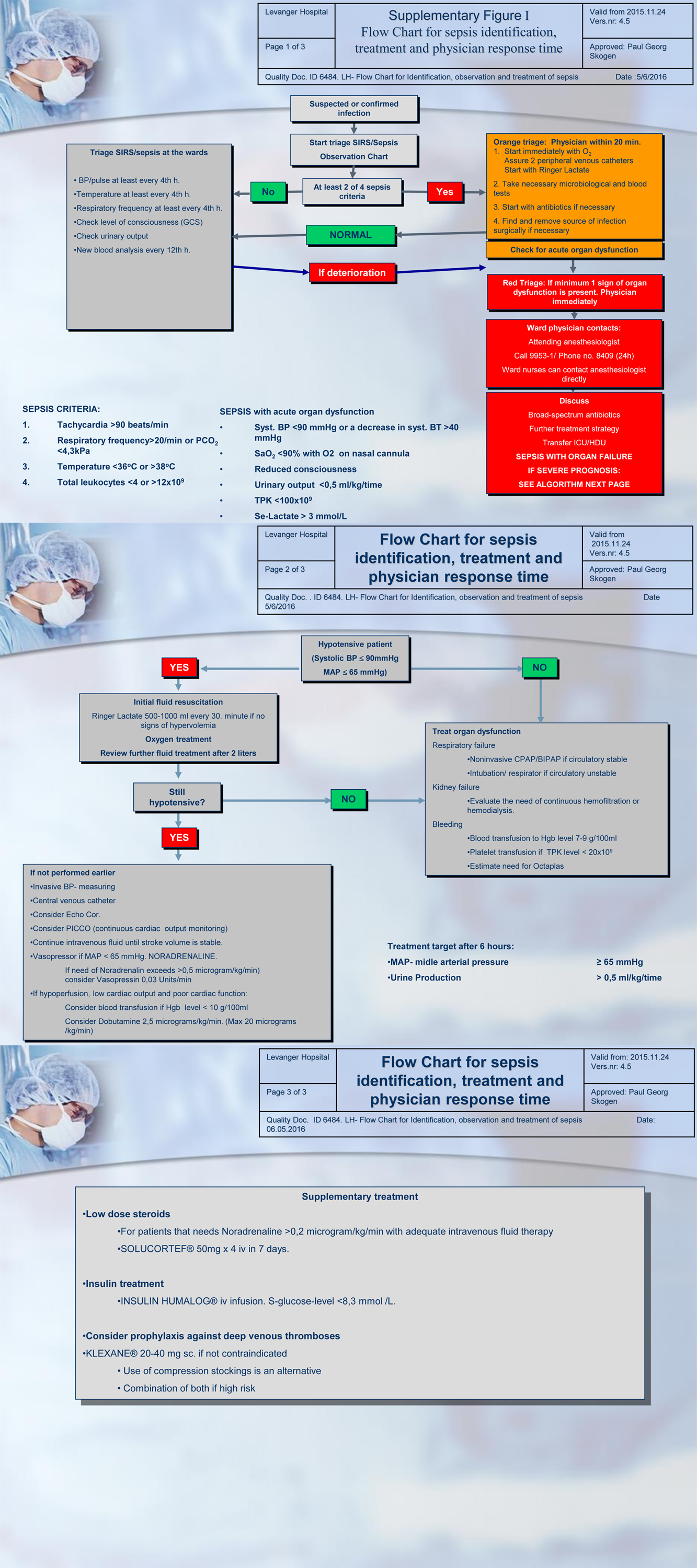

Supplement: Additional file 1: — Flow chart for sepsis identification, treatment and physician response time. (TIF 11739 kb) [file 13054_2016_1423_MOESM1_ESM.tif]
