# Supplementary material for: Early identification of sepsis in hospital inpatients by ward nurses increases 30-day survival
Source: Crit Care. 2016 Aug 5;20:244. doi: 10.1186/s13054-016-1423-1 (PMC4974789; doi:10.1186/s13054-016-1423-1)
Supplement: Additional file 2: — Detailed bloodstream infection (BSI) categorization in the pre-intervention and post-intervention group. (DOCX 13 kb) [file 13054_2016_1423_MOESM2_ESM.docx]

| Supplementary Table 1. Detailed Blood Stream Infection (BSI ) categorization in the pre and post intervention group (N=881). | | |
| --- | --- | --- |
|  | Control  (n=472) | Intervention  (n=409) |
| ***Microbes*** | **N (%)** | **N (%)** |
| Escherichia coli | 175 (37.8) | 143 (35.0) |
| Other enterobacteria^1^ | 56 (11.9) | 68 (16.6) |
| Other aerobic gram-negative bacteria^2^ | 22 (4.6) | 16 (3.9) |
| Anaerobic bacteria^3^ | 16 (3.4) | 10 (2.4) |
| Mixed bacterial or fungal infection^4^ | 31 (6.6) | 31 (7.9) |
| Streptococcus pneumoniae | 56 (6.6) | 27 (6.6) |
| Staphylococcus aureus | 43 (9.1) | 54 (13.2) |
| Betahemolytic streptococci | 24 (5.1) | 13 (3.2) |
| Enterococcus spp | 26 (5.5) | 19 (4.7) |
| Other gram positive bacteria^5^ | 23 (4.9) | 28 (6.9) |
| Chi-square between groups p 0.029 |  |  |
| ^1^Klebsiella spp, Proteus spp, Enterobacter spp, other Enterobacteriaceae. ^2^Pseudomonas spp, other gram-negative bacteria, Haemophilus influenzae, Neissera meningitis. ^3^ Anaerobe gram negative bacteria, anaerobe gram-positive bacteria. ^4^Mixed gram-negative aerobic and anaerobic bacteria, mixed gram-positive bacteria, other bacterial infections, mixed bacterial and fungal infections, Candida spp. ^5^ Viridans group streptococci, coagulase-negative staphylococci, Listeria monocytogenes. | | |
