# Supplementary material for: Early identification of sepsis in hospital inpatients by ward nurses increases 30-day survival
Source: Crit Care. 2016 Aug 5;20:244. doi: 10.1186/s13054-016-1423-1 (PMC4974789; doi:10.1186/s13054-016-1423-1)
Supplement: Additional file 3: — Sensitivity analysis for further potential confounders in the association between intervention and survival in all patients with BSI. (DOCX 12 kb) [file 13054_2016_1423_MOESM3_ESM.docx]

| Supplementary Table 2. Sensitivity analysis for further potential confounders in the association between intervention and survival in all patients with BSI (n=881) | | | | |
| --- | --- | --- | --- | --- |
|  | Age categories ^a^ | Immuno-suppression | Infection focus | Place of acquisition |
| Odds Survival 7 days | 1.7 (0.8–3.4) | 1.7 (0.8–3.4) | 1.5 (0.7–3.1) | 1.6 (0.8–3.3) |
| Odds survival 30 days | 2.1 (1.3–3.4) | 2.1 (1.3–3.5) | 2.0 (1.2–3.3) | 2.1 (1.3–3.5) |
| All supplementary adjustment models (except ^a^) are run in model 4 adjusted for age, sex, functional status, Charlson comorbidity index (CCI) and SOFA score. In adjustment model^a^ continuous age is replaced by categories of age (<65, 65-80,>80). | | | | |
