# Supplementary material for: Early identification of sepsis in hospital inpatients by ward nurses increases 30-day survival
Source: Crit Care. 2016 Aug 5;20:244. doi: 10.1186/s13054-016-1423-1 (PMC4974789; doi:10.1186/s13054-016-1423-1)
Supplement: Additional file 4: — Baseline characteristics of patients admitted with the first incident of bloodstream infection. (DOCX 13 kb) [file 13054_2016_1423_MOESM4_ESM.docx]

| Supplementary Table 3. Baseline characteristics of patients admitted with first incident of Blood Stream Infection (BSI) (N=738) | | | |
| --- | --- | --- | --- |
|  | Control  (N=405) | Intervention (N=333) | p |
| **Variables** | **N (%)** | **N (%)** |  |
| **Age** |  |  | 0.458 |
| <65 years | 126 (31.1) | 106 (31.8) |  |
| 65–≤ 80 years | 126 (31.1) | 115 (34.5) |  |
| >80 years | 153 (37.8) | 112 (33.6) |  |
| **Female** | 198 (48.3) | 179 (52.3) | 0.269 |
| **Place of Acquisition** |  |  | 0.005 |
| Community Acquired | 203 (49.5) | 129 (37.7) |  |
| Health Care Acquired | 158 (38.6) | 167 (48.8) |  |
| Hospital Acquired | 49 (11.9) | 462 (13.5) |  |
| **Functional status** |  |  | <0.001 |
| Independent | 239 (58.7) | 239 (68.9) |  |
| Partly Independent | 125 (30.7) | 59 (17.2) |  |
| Dependent | 43 (10.6) | 44 (12.9) |  |
| **Charlson Comorbidity Index (CCI)** |  |  | 0.089 |
| 0 | 119 (29.0) | 81 (23.7) |  |
| 1-2 | 164 (40.0) | 131 (38.3) |  |
| ≥3 | 127 (31.0) | 130 (38.0) |  |
| **SOFA score severe organ failure** | 81 (19.8) | 105 (30.7) | 0.001 |
| **Infection focus** | 361 (88.1) | 289 (84.5) | 0.157 |
| **Antibiotic started before admission (yes)** | 52 (12.7) | 57 (16.7) | 0.122 |
| **Immunosuppressant use (yes)** | 55 (13.4) | 66 (19.3) | 0.029 |
