# Supplementary material for: Early identification of sepsis in hospital inpatients by ward nurses increases 30-day survival
Source: Crit Care. 2016 Aug 5;20:244. doi: 10.1186/s13054-016-1423-1 (PMC4974789; doi:10.1186/s13054-016-1423-1)
Supplement: Additional file 5: — Odds of surviving 7 and 30 days in the first episode of sepsis in the pre-intervention group compared to the post-intervention group. (DOCX 12 kb) [file 13054_2016_1423_MOESM5_ESM.docx]

| Supplementary Table 4. Odds of surviving 7 and 30 days in first incident of sepsis (n=738 ) in pre-intervention group compared to post-intervention group (reference group) | | | | |
| --- | --- | --- | --- | --- |
|  | Model 1 | Model 2 | Model 3 | Model 4 |
|  | OR (95% CI) | OR (95% CI) | OR (95% CI) | OR (95% CI) |
| Survival at 7 days | 2.0 (0.9–4.4) | 2.0 (0.9–4.4) | 2.0 (0.9–4.4) | 2.4 (1.0–5.3) |
| Survival at 30 days | 2.2 (1.3–3.7) | 2.2 (1.3–3.8) | 2.3 (1.3–4.0) | 3.6 (1.9–6.9) |
| Model 1: crude model Model 2: Model 1+ Adjustments for age and sex. Model 3: Model 2 + functional status, place of acquisition and Charlson Comorbidity Index (CCI). Model 4: Model 3 + SOFA score | | | | |
